# The Microbiota‐Gut‐Brain Connection: A New Horizon in Neurological and Neuropsychiatric Disorders

**DOI:** 10.1111/cns.70593

**Published:** 2025-09-04

**Authors:** Md. Faysal, Mehrukh Zehravi, Baishakhi Sutradhar, Md Al Amin, Thukani Sathanantham Shanmugarajan, Uppuluri Varuna Naga Venkata Arjun, Susithra Ethiraj, Akiladevi Durairaj, Girija Dayalan, Shaik Khadeer Ahamad, Safia Obaidur Rab, Kannan Raman, Talha Bin Emran

**Affiliations:** ^1^ Department of Pharmacy, Faculty of Health and Life Sciences Daffodil International University Dhaka Bangladesh; ^2^ Department of Clinical Pharmacy, College of Dentistry & Pharmacy Buraydah Private Colleges Buraydah Saudi Arabia; ^3^ Department of Microbiology Gono University (Bishwabidyalay) Dhaka Bangladesh; ^4^ Department of Pharmaceutics, School of Pharmaceutical Sciences Vels Institute of Science, Technology and Advanced Studies (VISTAS) Chennai Tamil Nadu India; ^5^ College of Pharmacy Sri Venkateswara University Chennai Tamil Nadu India; ^6^ Department of Clinical Laboratory Sciences, College of Applied Medical Science King Khalid University Abha Saudi Arabia; ^7^ Department of Pharmacology St. Johns College of Pharmaceutical Sciences & Research Idukki Kerala India

**Keywords:** gut dysbiosis, microbiota‐gut‐brain axis, neuroinflammation, neurological disorders, neuropsychiatric disorders, probiotics

## Abstract

**Introduction:**

The microbiota‐gut‐brain axis (MGBA), a complex two‐way connection between the gut microbiota and the brain, has become a key regulator of neurological and neuropsychiatric disorders. Neurological disorders and gut microbiota dysbiosis are linked to these diseases. Changes in gut microbiota can lead to neurotransmitter imbalances, oxidative stress, and neuroinflammation. Gut dysbiosis may contribute to the development of diseases such as depression, autism, schizophrenia, bipolar disorder, Parkinson's disease, Alzheimer's disease, dementia, multiple sclerosis, epilepsy, anxiety, and autism spectrum disorders through immunological regulation, neuroinflammation, and neurotransmitter metabolism changes.

**Method:**

This review systematically sourced articles related to microbiota gut brain axis, neurological disorders, neuropsychiatric disorders and clinical studies from major medical databases, including Scopus, PubMed, and Web of Science.

**Results:**

This review explores the molecular processes underlying MGBA interactions, including vagus nerve signaling, systemic immunological responses, and metabolites produced by microorganisms. The discussion explores the potential of microbiome‐targeted treatments like fecal microbiota transplantation, probiotics, and prebiotics as effective treatment methods. The comprehension of the MGBA can revolutionize neurology and psychiatry, introducing innovative diagnostic and therapeutic approaches. Multiple elements, including diet, metabolism, age, stress, and medications, shape the human gut microbiota, and intestinal imbalances can lead to CNS diseases. The MGBA interacts with gut bacteria, and gut dysbiosis is associated with neurological disorders.

**Conclusions:**

The review demonstrates the correlation between gut microbiota and neurologically associated diseases, highlighting its importance in neurogenesis, mental development, emotions, and behaviors. MGBA, mediated by microbial metabolites, affects brain function and neuroinflammation. Interventions like fetal microbiota transplantation, probiotics, and prebiotics can improve microbial balance, but more clinical research is needed.

## Graphical abstract

The microbiota‐gut‐brain axis (MGBA) is a crucial regulator of neurological and neuropsychiatric disorders, with gut microbiota dysbiosis linked to various diseases. Gut microbiota changes can lead to neurodegenerative diseases like depression, autism, schizophrenia, Parkinson's, Alzheimer's, dementia, multiple sclerosis, epilepsy, anxiety, and autism spectrum disorders. Understanding the MGBA can revolutionize neurology and psychiatry, introducing innovative diagnostic and therapeutic approaches.

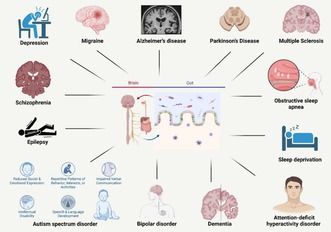

## 1 Introduction

The human stomach contains 500 million neurons, while the brain contains 100 billion. The gut affects brain function through neurons, blood circulation, and lymphatic pathways, and is linked to our central nervous system (CNS). The gut microbiota is another essential element in the relationship between the gastrointestinal (GI) system and the brain. Trillions of bacteria make up this intricate ecosystem designated as the microbiota. The gut flora is affected by numerous factors, including nutrition, metabolism, age, location, sleep, temperature, seasons, stress, and drugs. Both physical and mental well‐being, along with the prophylaxis and management of several diseases, depend on the gut microbiota's compositional structure being in the proper balance. The microbiota‐gut‐brain axis (MGBA), a focus of growing clinical interest, refers to the interplay among these various brain and gastrointestinal tract (GIT) regions, including the microbiota. Modifications in gut microbiota are being investigated for the increased prevalence of neurological, mental, and sleep problems to identify the mechanisms of action and develop treatment plans [1, 2]. MGBA has a role in the pathophysiology of neurodegenerative diseases (NDs). It demonstrates structural and functional changes in the GI and blood–brain barrier (BBB) interfaces in ND patients, as well as disruptions in gut microbiota and its metabolomic landscape. Recent developments in dietary and pharmaceutical treatments aim to modify the MGBA for ND treatment, improving our understanding of the complex mechanisms underlying ND [3]. Gut bacteria regulate neurotransmitters, influencing mental health and brain function. It demonstrates therapeutic approaches targeting the gut microbiota to treat NDs. It also combines clinical research, neuroscience, and microbiology, highlighting the complex interactions between the CNS and gut microbiota [4].

The gut microbiota is a diverse ecosystem with genetic traits affecting host health. The human GI system, home to 100 trillion bacteria, acts as a “virtual organ” affecting host health, fitness, and phenotype [5]. The microbiome, comprising all microorganisms within a habitat, and microbiota, a diverse group of bacteria in human anatomy, significantly impacts the host's health through their intricate connections [6]. Intestinal dysbiosis is increasingly linked to unhealthy microbiota and the pathophysiology of extra‐intestinal and gut‐related diseases [7]. The gut microbiota significantly influences a person's health, with pathogenic microorganisms, also known as pathobionts, potentially causing negative consequences [8]. Furthermore, the gut microbiota significantly influences the host's vital physiological processes, including intestinal barrier integrity, immune system function, metabolic and nutritional homeostasis, and cerebral activity [9]. The gut microbiota plays a crucial role in regulating vital processes for host homeostasis, including cardiovascular health, metabolism, and immunological and inflammatory responses [10]. Human gut microbiota exhibits significant diversity, but a third remains identical. Factors like diversity changes, and commensal loss can cause dysbiosis [11]. Research indicates that gut microbiota impacts neuropsychiatric health and GI problems. Understanding host‐microbiota communication pathways, particularly the MGB axis network, can help regulate symbiosis and dysbiosis [12]. The gut‐brain axis is a crucial communication channel between the gut and the CNS, impacting health and disease. The gut microbiota can alter this relationship to maintain homeostasis [13]. The ENS, a branch of the autonomic nervous system, regulates gut functions like digesting, bile secretion, and immune response, involving motor, sensory, and interneurons from the myenteric and submucosal ganglia [14]. Research indicates gut microbiota can interact with the CNS through neuromodulatory metabolites and neurotransmitters like glutamine, histamine, tryptophan, 5‐HT, GABA, and SCFAs [13]. Enterochromaffin cells release serotonin (5‐HT) in response to stimuli, influenced by GI microbiota. Microbes like *Klebsiella*, *Escherichia coli* , *Streptococcus*, *Lactobacillus*, and *Lactococcus* produce serotonin, while other bacteria reduce host access to luminal tryptophan [15]. GABA, an inhibitory neurotransmitter, can be converted by the host or microorganisms like *Lactobacillus* species and *Escherichia* species, raising GABA levels in the ENS [16]. The brain's functioning and emotional regulation rely on a healthy microbiome, and preventing dysbiosis, a gut organism imbalance, is crucial for reducing potential health risks [17]. The intestinal microbiome's bacteria comprised around 100 g of the wet weight of the intestinal colon, which was between 200 and 250 g of colonic component [18]. Archaebacteria microorganisms, microorganisms found in anaerobic environments, are crucial to the intestinal microbiota, generating energy through methanogenesis, a process that produces methane from various substrates [19]. Different bacteria colonize different parts of the GIT due to factors such as intestinal peristalsis, oxygen, pH, and nutritional availability [6, 20]. Intestinal motility can be influenced by various diseases, abdominal cavity surgery, GIT changes, gastric acid secretion, or resection of a large intestine fragment [21, 22]. This review highlights that gut microbiota disruptions cause neurodegenerative and neuropsychiatric diseases, causing inflammation, OS, and neurotransmitter dysregulation. Microbiome interventions like probiotics and transplantation can restore balance.

## 2 Both Extrinsic and Intrinsic Factors Influence the Gut Microbiota and Neuropsychiatric Diseases

Certain extrinsic and intrinsic factors influence health and neuropsychiatric status, such as dietary choices, medication use, and lifestyle choices. In conjunction with heredity, neurotransmitter dysregulation, and inflammation, lifestyle choices also play an essential part in the relationship involving gut microbiota and the brain. They are part of the multifactorial etiology of psychiatric diseases. Physical activity is widely recognized to improve cognitive performance and reduce the likelihood of cognitive impairment. Lifestyle choices like oral hygiene, nicotine use, and sleep deprivation significantly impact the progression of gut dysbiosis and brain‐related diseases [23, 24, 25, 26]. Dietary variables significantly impact health and wellness, as human eating patterns are influenced by societal, cultural, and religious factors. Dietary choices can significantly impact both individual and public mental health, as they influence brain development and cognitive ability. Diet can either maintain homeostasis or significantly contribute to disease development. By over‐representing bacteria that express lipopolysaccharide (LPS), a high‐fat diet (HFD) primarily made up of saturated and/or trans fats appears to alter the microbiota. This results in increased concentrations of LPS within the host's circulatory system, a pro‐inflammatory condition, and decreased synaptic plasticity [27, 28, 29]. A healthy intestinal microbiota, producing immunomodulatory materials like short‐chain fatty acids (SCFAs), regulates the gut‐associated lymphoid tissue (GALT) and prevents immune‐driven NDs [30, 31]. The Mediterranean diet, rich in nuts, legumes, whole grains, and fresh fruits and vegetables, can significantly influence the formation of SCFAs. Consuming meat, poultry, and fish in moderation gives probiotic bacteria fermentable substrates. The Mediterranean diet lowers clinical depression risk due to vitamin B content and neurotransmitter production, regulating mood and behavior through monoamine turnover [32]. Numerous biological and chemical elements also affect the neuropsychiatric state. From a biological perspective, some physiological conditions, such as being overweight, diabetes, reduced pulmonary functionality, and urological and genital disorders, might disrupt mental health [33, 34]. Certain chemicals, like medications or antibiotics, can significantly impact the onset and progression of mental diseases [35].

A meta‐analysis reveals significant changes in taxonomy and metabolic capacity of gut microbiota regulation, with correlations with gut operational changes due to frequent drug use. The misuse or abuse of certain chemicals can significantly alter resistome profiles, leading to the identification of 19 out of 41 drugs as having microbiological characteristics. Laxatives, metformin, antibiotics, and proton pump inhibitors exhibited the most significant correlations between the gut microbiome and cerebral function [36]. Among drugs, antibiotics are likely the most commonly utilized drugs for various ailments. Antibiotics are effective in combating infectious diseases but can also alter the gut microbiota and microbiome, potentially leading to immunological dysregulation. Antibiotic use disrupts the balance between commensal populations, altering the gut microbiota's makeup and function, leading to long‐term adverse effects on the host [37, 38]. While short‐term antibiotic administration is linked to impaired cognitive function [39, 40], high dosage or prolonged antibiotic administration can cause significant changes or irreversible harm at the intestinal and brain levels [41, 42]. A study on adult mice found that antibiotic treatment alters microbial communities and colon and plasma metabolite profiles, disrupts neuronal signaling molecules, and impairs object recognition. The study also found that certain antibiotics, such as ampicillin, bacitracin, and meropenem, impair brain function [40]. Antibiotic treatments disrupt the diversity of gut microbes; for example, Proteobacteria and Bacteroidetes rapidly replace Firmicutes and Actinobacteria classes. This phenomenon is linked to a lower level of SCFAs in the colon, further linked to neurological and brain dysfunctions [30, 38, 43, 44]. When used to treat GI and other infections, some broad‐spectrum antibiotics are capable of crossing the BBB and partially entering brain tissues, which can lead to neurological decline [43, 45]. Furthermore, antibiotic‐induced intestinal dysbiosis in early life can negatively impact neurological development in later life but can also lead to the development of diseases like diabetes, obesity, allergies, and irritable bowel syndrome (IBS) [30, 46, 47]. Additionally, chronic antibiotic use leads to altered neuromodulator levels in the gut‐brain axis, resulting in cognitive impairments, altered metabolic pathways, and decreased expression of neurotrophic factors [46, 48]. Clinical data show that antibiotic therapies can cause disruptions in the gut microbiota in various pathways, ranging from temporary to irreversible changes [42]. Furthermore, clinical studies show that antibiotic usage leads to gut microbiota changes, resulting in impaired object recognition, memory retention, and decreased hippocampal neurogenesis [49, 50].

## 3 MGB Axis in Neuropsychiatric Disorders

**FIGURE 1:**
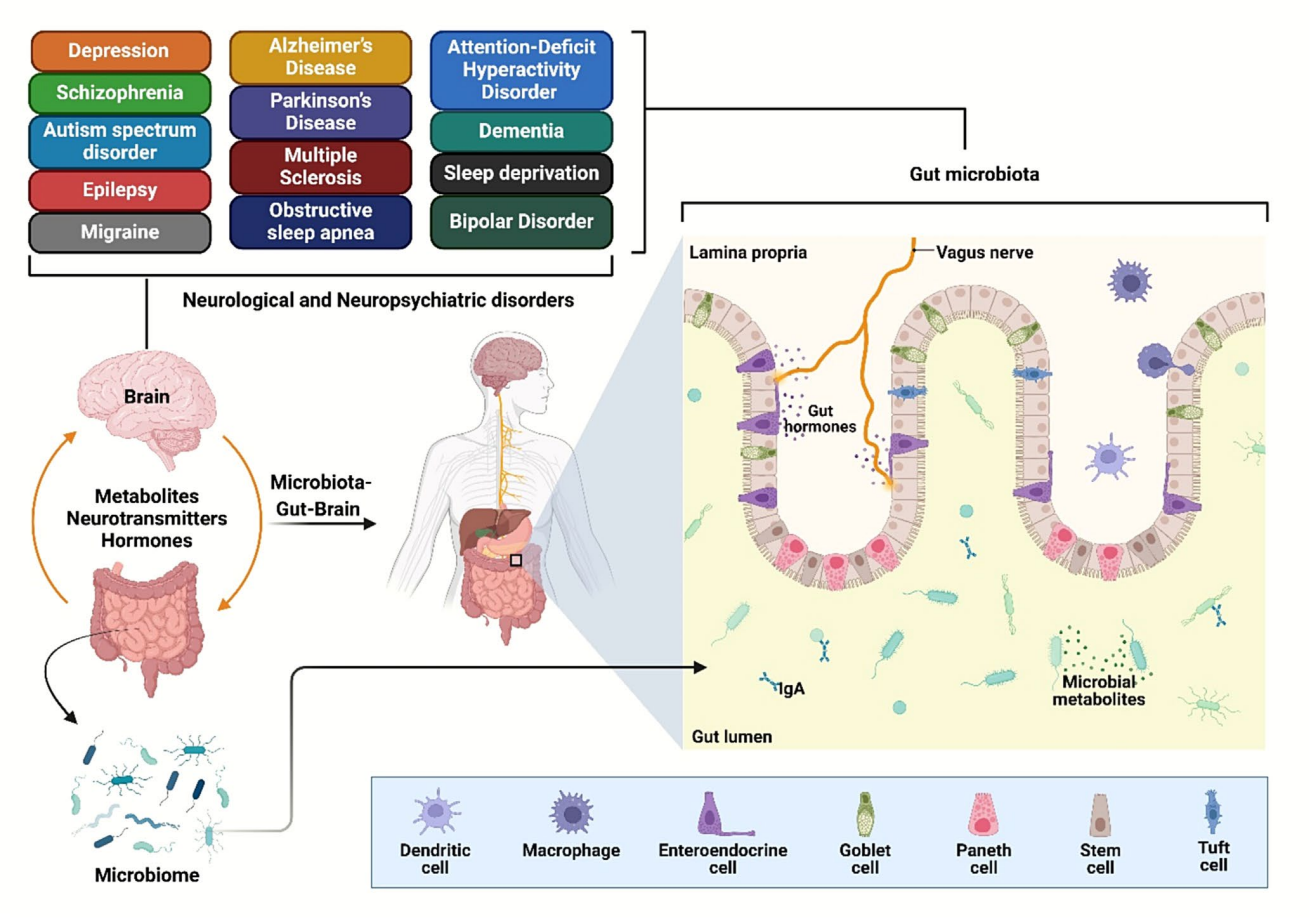
The MGBA is a complex communication network between the gut microbiota and the CNS, affecting brain function and behavior. Mutations in the gut microbiota may be a factor in neurological and neuropsychiatric disorders like ASD, AD, PD, and anxiety.

The MGBA (Figure 1), a two‐way intercommunication between the brain and intestinal microbiota, has been associated with neuropsychiatric health. The gut microbiota may influence behaviors related to psychiatry and CNS diseases, and targeting the MGBA could be a potential treatment strategy [51]. The majority of adult and pediatric neuropsychiatric disorders are thought to be multifactorial diseases brought on by environmental variables in genetically predisposed people [52]. The potential gut microbiota changes that may impact the onset of various neuropsychiatric diseases and their expressions, as well as their intricate gene–environment interactions. Ecological risk determinants and genetic loci interact to produce epigenetic modifications, leading to high phenotypic variation in neuropsychiatric diseases without nucleotide sequence changes [53, 54]. These epigenetic changes can result in DNA methylation and other alterations that immediately affect histone proteins and alter the transcription of genes [55]. Dysbiosis and modified intestinal permeability are pathogenic due to their ability to allow neurotoxic chemicals into the circulatory system, abnormally activating the immune system [56]. Gut microbiota directly impact the brain by regulating the immune system, maintaining a low level of immune activation due to continuous support for the immune system. This interaction strengthens the immune barrier and the microorganisms' containment [57, 58]. Neuropsychiatric diseases like major depressive disorder (MDD), schizophrenia (SCZ), and autism spectrum disorder (ASD) [59], multiple sclerosis (MS), Parkinson's disease (PD) and Alzheimer's disease (AD), or epilepsy may have changes in the composition of their gut microbiota and MGBA [60, 61]. Individuals with this condition often experience a higher incidence of gastrointestinal functional discomfort, including heartburn, diarrhea, constipation, abdominal discomfort, and irritable bowel syndrome [62, 63].

## 4 The Microbiota‐Gut‐Brain Axis in Neuropsychiatric and Neurological Disorders

### 4.1 Depression

Depression, specifically MDD, often leads to fatal results due to various functions such as food intake, mood, cognitive function, psychomotor performance, and sleep [64, 65]. Depression affects around 264 million individuals [66]. A persistent sense of melancholy and indifference that lasts for at least 2 weeks is the hallmark of depression. Common mental disorders can be caused by various psychological, social, and biological factors, including significant life changes, familial issues, long‐term health issues, or addiction [67, 68]. Significant depression is a prevalent mental disorder with a complex etiology, characterized by abnormalities in the prefrontal cortex and hippocampus. While depression is believed to be triggered by stress, depression patients and animal models of the disease have a malfunction of the hypothalamic–pituitary–adrenal (HPA) axis [69]. Stress exposure is frequently the basis for animal models of depression [70]. Additionally, about two‐thirds of individuals with MDD experience anxiety as a main feature or a comorbidity [69, 71]. Anxiety‐like behavior frequently coexists with depression‐like behavior in animal models [72]. A study [73] investigating the profile of SCAFs [74] found that 116 women aged 52.0 (±4.7) years, with 40.52% of them recognizing depression, had a higher content of isocaproic acid and a lower level of propionic acid than healthy participants. The certainty of whether SCAFs contribute to the depressed phenotype is uncertain due to the small group size. The composition of the intestinal microbiota has been linked to various personality and behavior traits, such as anxiety and depression [75]. Depression is a condition characterized by changes in cerebral morphology, abnormal neuronal circuitry, stress hormones, inflammatory markers, and disruption of neurotransmitter systems, necessitating multimodal diagnosis and therapy. Brain imaging studies have revealed anatomical alterations in the hippocampus, amygdala, and PFC, as well as changes in neuroplasticity‐related proteins [76]. The gut microbiota significantly influences the pathophysiology of depression. Changes in its makeup can set off inflammatory reactions that impact behavior via the HPA and other mechanisms. Studies suggest that changes in the gastrointestinal microbiota may increase the production of microbial lipopolysaccharides, leading to inflammatory reactions and potentially causing depression symptoms. Furthermore, studies have revealed that the diversity and makeup of gut bacteria vary between depressed and nondepressed individuals. Dysbiosis affects mood and behavior in individuals with depression, affecting their gut microbiota's abundance of Firmicutes, Bacteroides, and Actinobacteria, and influencing NDs linked to depression [77]. The gut microbiota and the brain are strongly correlated in MDD. Fatigue can disrupt gut microbiota balance, causing dysbiosis with lower SCFA levels and increased proinflammatory cytokines, particularly interferon‐gamma and IL‐6. The inflammatory condition can lead to the deterioration of the gut's integrity, facilitating the migration of bacteria more easily. Inflammatory cytokines increase, activating indoleamine 2 and 3‐dioxygenase, disrupting protective metabolites like kynurenic acid, and disrupting the kynurenine pathway. The pathway's inflammatory cytokines and toxic metabolites may weaken the blood–brain barrier (BBB), leading to microglial activation, astrocyte loss, and increased inflammation in brain tissue. The sequence of events may exacerbate behavioral diseases like anxiety and MDD. Probiotics and prebiotics can improve gut microbiota and intestinal barrier integrity, reducing inflammation, harmful metabolites, and BBB permeability through indirect means [78].

Fecal microbiota transplant (FMT) has the potential to reduce symptoms of mental disease. When a person with a healthy microbiota receives bacteria from a person with a distressed microbiota, they may experience symptoms. Gut microbiome significantly influences mental disease, with FMT from healthy donors often alleviating symptoms for three to 6 months [79]. MGBA can induce and alleviate depression symptoms, demonstrating its reciprocal effect on depression. The study found that rats exposed to high frequencies of “depression‐related microbiomes” exhibited depressive‐like behaviors compared to their control group [80]. However, Wouw et al. found that administering mice a mixture of SCFAs such as propionate, butyrate, and acetate may reduce stress‐induced depressive‐like behaviors [81]. A proteomics study using isobaric tagging found significant protein alterations in a mouse model of depression caused by FMT from depressed individuals [82]. Li et al. revealed that genetic modifications in mice can increase anxiety and depression symptoms, possibly due to disrupted MGBA, indicating a potential underlying cause (Table 1) [83]. Moreover, Huang et al. comparing the olfactory bulb (OB) of germ‐free mice with pathological and healthy microbiota found 367 differentially identified proteins in the OB. It demonstrates the importance of CREB signaling, which affects OB axonogenesis under microbiota conditions. The findings add to the understanding of gut bacteria's role in mental diseases and offer a fresh perspective on the complex processes underlying OB dysfunction in depression. The research contributes to the understanding of depression and its impact on the brain [85]. Furthermore, the gut‐brain axis links gut microbiota to neuropsychiatric diseases, but little research has been done on how antidepressant medication affects gut flora. A study using fluoxetine found that stress increases pathogen abundance and decreases bacterial diversity, but fluoxetine inhibits these changes. Strong associations between gut microbiota and anxiety and depressive behaviors were also revealed [88].

**TABLE 1:** Recent preclinical studies have explored the impact of gut microbiota on depression and anxiety.

Animals used	Model and/or treatment	Behavioral test	Outcomes	References
C57BL/6J mice	CUMS model FMT	Sucrose preference test Open‐field test Elevated plus maze Forced swim test	Microbial dysbiosis, including Lactobacillus depletion and Akkermansia enrichment, was linked to neuroinflammation, while FMT from CUMS donors caused anxiety and despair in mice.	[83]
Lewis rats	FMT	Light–dark test Sucrose preference test Elevated plus maze test Open‐field test	FMT from healthy rats reduced anxiety‐like behavior in rats with incomplete unilateral cervical SCI and prevented dysbiosis caused by SCI.	[84]
GF mice	CUMS and OB model FMT from human donors	Open field test Tail suspension test Forced swim test Olfaction behavior test	FMT from MDD patients caused depressive‐like behaviors in mice, while gut bacteria caused unique molecular alterations in neurogenesis and pathways.	[85]
GF NIH Swiss mice	FMT from human donors	Light–dark test Step‐down test	Mice receiving FMT from patients with IBS displayed a taxonomically comparable microbial makeup and various metabolomic patterns of serum based on their microbiota profiles grouped based on human donors.	[86]
Sprague–Dawley rats	Social defeat model FMT	Forced swim test Social interaction test	Rats treated with FMT from SL/susceptible rats exhibited depression‐like behaviors, higher microglial density, and IL‐1β expression in the ventral hippocampus compared to nonstressed rats.	[87]
C57/6 mice	CUMS Fluoxetine (12 mg/kg) p.o.	Tail suspension test Sucrose preference test Elevated plus maze test Open field test	Stress leads to low bacterial diversity, a simplified network, and increased pathogens. Fluoxetine reduces stress‐induced GM alterations, and GM is linked to anxiety and depressive behaviors.	[88]
Swiss mice	*Lactobacillus plantarum* 286 or *Lactobacillus plantarum* 81 (109 CFU) p.o. for 30 days	Open field test Forced swim test Plus maze‐discriminative avoidance test	*Lactobacillus plantarum* 286 and 81 treatments did not affect learning, memory, or locomotor activity, but they caused behaviors resembling anxiolytics and antidepressants in mice.	[89]
GF rats	FMT from human donors	Forced swim test Sucrose preference test	FMT from depressed patients caused depressive‐like behaviors in recipient rats, decreased neurotransmitter levels, and improved HPA axis hyperfunction.	[90]
GF and PF Kunming mice	FMT from human donors	Open field test *Y*‐maze test Tail suspension test Forced swim test	Mice lacking GM displayed depressive‐like behaviors, while GF recipient mice from MDD patients exhibited depression‐like behaviors due to FMT and GM‐influenced host metabolism.	[91]
BALB/c and Swiss	Acute restraint stress	Tail suspension test	The presence of *Lactobacillus rhamnosus* JB‐1 in BALB/c mice, unlike Swiss Webster mice, has been linked to decreased depressive‐like behavior and accelerated recovery.	[92]
C57BL/6 mice	Chronic social defeat stress	Open field test Three‐chambered sociability test Aggressor interaction test	Defeated mice displayed decreased functional diversity, altered dendritic cell activation, and elevated IL‐10 + T regulatory cells.	[93]

### 4.2 Schizophrenia

Cognitive, emotional, and occupational problems are all part of the complex disease that is schizophrenia [94]. SCZ‐positive adults face a high risk of premature death due to viral, metabolic, and cardiovascular diseases. The average potential life lost for people with SCZ in the US is 28.5 years [95]. The cause of schizophrenia involves the interaction between prenatal and postnatal ecological influences and genetic predisposition. A prenatal microbial infection was associated with a markedly elevated chance of developing schizophrenia [96]. Interestingly, intestinal barrier failure, bacterial translocation, and GI comorbidities are more common in people with schizophrenia [97]. In addition to cognitive impairments, GF mice exhibit amplified stereotypical, recurring, and locomotor responses [98, 99]. Cognitive impairment is common in schizophrenia, but hyperactivity and extreme stereotypy are common in animal models and are believed to be positive symptoms in humans [100]. Additionally, one of the negative signs of schizophrenia is social [101]. GF mice exhibit less social stimulus and a desire for novel social interactions. Bacterial colonization postweaning effectively reverses social preference deficit but not social cognition, confirming microbiota's role in influencing social choice [102]. Crucially, FMT from GF mice to people with schizophrenia has been shown to induce some behavioral alterations associated with schizophrenia. Mice transfected with schizophrenia‐related microbiota showed increased startle responses, decreased anxiety, depressive behavior, increased locomotor activity, and elevated glutamate and GABA levels in the hippocampus [103], and changes in glycerophospholipid and fatty acyl metabolism [104] were linked to animal behavioral changes. In a different study, FMT from untreated schizophrenia patients to PF mice caused dysregulated tryptophan‐kynurenine metabolism and aberrant behaviors comparable to schizophrenia, including psychomotor hyperactivity and cognitive impairment [105]. *Streptococcus vestibularis* is the only bacterium that has recently been transfected into mice that had antibiotic‐induced microbiota loss. Numerous patients with schizophrenia have this bacterium in their guts, and it has 11 gut‐brain modules that are involved in the synthesis and breakdown of multiple neurotransmitters, including glutamate and GABA. A *Streptococcus vestibularis* transplant resulted in hyperkinetic behavior, decreased social interaction, altered peripheral neurotransmitter levels, and altered gene expression related to inflammation and the immune system. Schizophrenic individuals exhibit decreased serum tryptophan concentrations and higher kynurenic acid levels, with the prefrontal cortex exhibiting a lower tryptophan level [106]. Schizophrenia's pathophysiology may be linked to gut microbiota and altered tryptophan‐kynurenine metabolism [105, 106]. A mouse model of schizophrenia showed significant variation in gut microbiome diversity due to a deletion of the metabotropic glutamate receptor 5. The study revealed that mice lacking mGlu5 showed reduced abundances of the Allobaculum genus and Erysipelotrichaceae family [107]. He et al. found the gut microbiome variations between healthy individuals and people with SCZ. The reports showed significant variation in the profusion of specific taxa among the control and schizophrenic groups. There were no appreciable variations in microbiological variety among high‐risk, ultra‐risk, and healthy controls [108]. The genera Lactobacillus and Prevotella and the orders Clostridiales, Lactobacillales, and Bacteroidales were notably higher in the ultra‐risk patients than in the other two groups. Similarly, there were no variations in the microbiome diversity indices among healthy controls and patients with schizophrenia [109]. Moreover, Nguyen et al. found the gut microbiota composition in schizophrenia patients with a prolonged disease duration. Proteobacteria exhibited a relative decline at the phylum level in comparison to the controls. Anaerococcus increased in schizophrenic individuals, while Clostridium, Sutterella, and Haemophilus decreased, despite a decrease in microbial diversity at the genus level. The study found that an increase in Ruminococcaceae prevalence was linked to a decrease in the intensity of negative schizophrenia symptoms [110].

Additionally, Li et al. observed a higher relative abundance of Actinobacteria and a reduced prevalence of Firmicutes at the phylum level. At the genus level, there was an increase in Eubacterium, unidentified Ruminococcus, Mogibacterium, Corynebacterium, Succinivibrio, Lactobacillus, and Collinsella. At the same time, Ruminococcus, Anaerostipes, Faecalibacterium, and Adlercreutzia showed a decrease in abundance among patients with schizophrenia. Succinivibrio and Corynebacterium were significantly associated with the intensity of schizophrenia manifestations, potentially offering novel biomarkers for schizophrenia diagnosis [111]. A human study reveals unique schizophrenia‐related bacteria correlate with changes in the right middle frontal gyrus volume, suggesting a possible link between gut microbiota and brain anatomy in schizophrenia [112]. Research shows gut flora's role in antipsychotic therapy, with initial microbial profile changes in rats after olanzapine administration [113, 114]. Davey et al. found decreased diversity in microbial composition, increased Firmicutes abundance, and decreased Bacteroidetes in individuals with weight gain, adipose tissue volume, and inflammatory and metabolic markers [114]. Moreover, Morgan et al. found that mice showed similar changes in gut microbiota and body weight gain after olanzapine administration, even when using adjusted P values compared to unadjusted P values. Olanzapine administration decreased microbial diversity, decreased Bacteroides' abundance, and increased Erysipelotrichia, Actinobacteria, and Gammaproteobacteria's abundance. The study revealed that the weight gain induced by olanzapine in GF mice is influenced by the presence of gut microbiota, without causing significant weight gain [115]. Numerous studies have explored the influence of gut flora on the weight gain resulting from risperidone. Risperidone therapy in rats resulted in a significant increase in body mass due to decreased energy expenditure and changes in gut flora. Firmicutes prevalence increased, while Bacteroidetes decreased [116]. Prolonged risperidone administration in juvenile patients led to increased BMI and decreased Bacteroidetes: Firmicutes ratio, with specific taxa linked to weight gain [117]. Risperidone treatment increased *Bifidobacterium* spp. and *Escherichia coli* in drug‐naïve individuals experiencing their first schizophrenia episode [118].

Ma and his colleagues found alterations in gut microbial composition linked to various antipsychotic medications [112]. Moreover, Zheng et al. found global microbial phenotypes were unaffected by sex or medicines [103]. A meta‐analysis of randomized trials reveals that antibiotics, probiotics, or prebiotics have minimal impact on schizophrenia symptoms [119]. In rats, antibiotic treatment was demonstrated to mitigate weight increase induced by olanzapine [113] and eliminate phencyclidine‐induced memory problems [120]. Research regarding the impact of probiotics on schizophrenia is limited. A randomized, placebo‐controlled experiment indicated that a 14‐week probiotic supplementation failed to enhance psychopathological manifestations in people with schizophrenia [121]. The group receiving probiotics was less prone to experiencing severe bowel movement issues, perhaps attributable to the immunomodulatory effects particular to probiotics [122].

A 12‐week trial showed that a combination of vitamin D and probiotics improved schizophrenia patients' overall scores, antioxidant levels, inflammation, and metabolic profiles. The study did not determine the treatment's impact on the gut microbiome, and the specific element responsible for the observed changes remains unclear [123]. A 4‐week trial found that *Bifidobacterium breve* A‐1 consumption reduced anxiety and depression in schizophrenia patients, but this effect was not linked to gut microbiome changes [124].

Guo et al. [125] demonstrated that inulin mitigated MK‐801‐induced schizophrenia‐like behavior in mice by altering gut microbiota formulation and anti‐inflammatory effects. In rats, administering the B‐GOS prebiotic formulation enhanced cognitive adaptability [126] and mitigated the weight gain produced by olanzapine [127]. The prebiotic supplementation was observed to enhance cortical neuronal responses to NMDA [126], augment cortical expression of NMDA receptor subunits [128], and boost hippocampal BDNF levels [129]. Schizophrenia is influenced by hypofunction of the NMDA receptor and reduced levels of BDNF. A double‐blind, placebo‐controlled crossover trial found that the B‐GOS prebiotic showed a beneficial cognitive effect in individuals with psychosis without significant changes in metabolic and immune system parameters [130]. Nagamine et al. found that prebiotic administration in schizophrenia patients, who were given chlorpromazine, led to weight gain and altered microbiota composition [131]. Moreover, Flowers et al. found that resistant starch supplementation in patients with atypical antipsychotic medications caused gut flora changes without affecting body weight [132]. The etiopathogenesis of SCZ is analyzed using biochemical and neuroimaging findings. Dopamine is primarily involved in the pathogenesis of depression, although it may also indirectly play a role [133]. The connections between other neurotransmitters should also be considered as sources of SCZ [134, 135]. The etiology of SCZ has been linked to immune/inflammatory processes [136]. Alarmins activate signaling pathways in inflammatory disorders, involving the glutamatergic system, with genes like neuregulin 1 and 13q33 activating NMDA receptors and amino acid oxidase [137]. The identification of specific genes supports the neurodevelopmental theory of SCZ and the role of the glutamatergic system in this process [138]. Positive symptoms of SCZ are characterized by a suppression of glutamatergic transmission and an increase in dopaminergic transmission in the mesolimbic region. The increasing evidence suggests that kynurenic acid (KYNA) may potentially influence both pathways [139]. KYNA, a nonselective antagonist of excitatory amino acid receptors, acts as an antagonist to the AMPA receptor and NMDA receptor complex. Studies reveal higher KYNA levels in SCZ patients, suggesting potential involvement in CNS physiology and pathophysiology. Variations in KYNA levels between ill and healthy individuals suggest its role in neurological and mental disorders [140].

The overall compositional structure of the SCZ gut microbiota was substantially different from that of the metabolic syndrome and healthy control participants. Common GI comorbidities in SCZ include Celiac disease, inflammatory bowel disease, and irritable bowel syndrome, which are immunological responses to gluten that damage the small intestine [141]. SCZ is linked to OS, persistent GI inflammation, and disruptions of the gut flora [110]. *Sellimonas intestinalis*, *Bilophila wadsworthia* , *Collinsella aerofaciens* , and *Flavonifractor plautii* were noticeably more prevalent in the SCZ gut microbiota. Still, *Veillonella rogosae* , *Ruminococcus lactaris* , *Ruminococcus bicirculans*, and *Faecalibacterium prausnitzii* were less prevalent [142]. Cordeiro et al. suggested probiotics and prebiotics can supplement the management of microbiome abnormalities in SCZ patients, highlighting connections between microbiome diversity and immunological and metabolic pathways [143]. Moreover, Nguyen et al. found that Clostridia, along with Proteobacteria, Firmicutes, and Clostridia taxa, showed significant differences at the phylum level in the South China Sea [110]. Lactobacilli levels may be linked to the course of SCZ and are correlated with the severity of symptoms [144]. Furthermore, immunological dysregulation, including changes in memory T cell function, could be a potential factor in the pathophysiological mechanisms of SCZ. A study found an inverse relationship between alpha diversity and a specific subset of memory T cells in SCZ patients, highlighting the intricate interconnections between the immune system and the microbiome [143]. Dysbiosis can worsen inflammation by increasing intestinal permeability, which is influenced by the kynurenine pathway, which converts tryptophan into kynurenine, a crucial amino acid. Disruption of this route has been linked to numerous mental diseases, with NMDAR hypofunction linked to SCZ, and kynurenate acting as a broad‐spectrum glutamate receptor antagonist [145].

### 4.3 Autism Spectrum Disorder

ASD is a neurodevelopmental disorder characterized by repetitive behavior, social difficulties, and difficulties with vocal and nonverbal communication. The interplay of the environment, immune system, genetics, and in utero influences is part of the pathogenesis [146]. Depression, anxiety, seizures, dysbiosis of the gut, and other GI issues are common concurrent pathologies in patients with ASD [147, 148, 149]. In contrast to control children, children with ASD had a considerably higher prevalence of GI symptoms. The range of estimates was 17% to 86%. GI disorders, when present, exacerbate neurobehavioral symptoms in autistic children [150], even though dysbiosis appears to be independent of the occurrence of GI symptoms in these individuals [151]. There has been evidence of a relationship between the intensity of GI symptoms and the intensity of symptoms associated with ASD [152]. Abdominal discomfort is a potential cause of violence, self‐harm, or sleep disorders in children with ASD [74]. ASD cooccurring with GI issues has been linked to polymorphisms found in a number of genes or the activity of enzymes, such as disintegrin and metalloproteases [153, 154, 155]. GF rodents exhibited increased recurrent burial behavior, stereotyped self‐grooming, and decreased social behavior, including decreased sociability and interaction with new partners [102, 156, 157]. GF mice's social behavior abnormalities improved after being recolonized with the usual gut microbiota [158]. A study found early‐life antibiotic use linked to higher ASD risk, but this association disappeared when shared familial environment and genetics were considered [159]. Changes in beneficial microbes led to a shift toward those producing spores, resistant to antibiotics, or producing neurotoxins, with lower levels of Prevotella, Corprococcus, Veilonellaceae, Bacteroidetes, Actinobacteria, and Proteobacteria [160].

**FIGURE 2:**
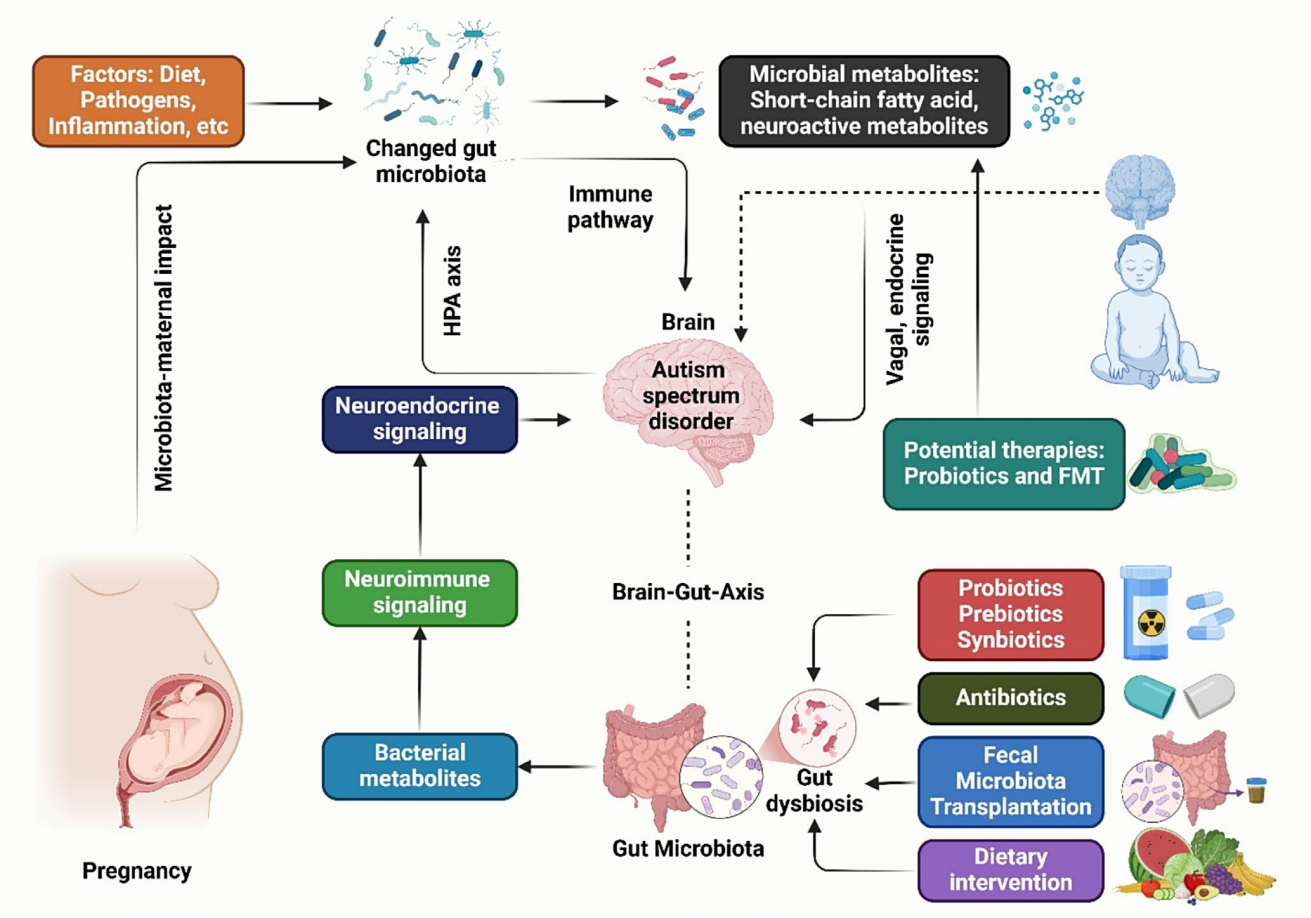
The MGBA is a key player in the pathophysiology of ASD, with behavioral symptoms and neurodevelopmental problems influenced by changes in gut permeability, immunological responses, microbial metabolites, and gut microbiota composition. Mechanisms connecting ASD to the MGBA include immune dysregulation, neuroinflammation, neurotransmitter imbalance, and stress response. Therapeutic interventions targeting the MGBA include probiotics, prebiotics, fetal microbiota transplantation, and nutritional measures like omega‐3 fatty acids.

The development of ASD may be linked to an imbalance in the proliferation of pro‐inflammatory *Clostridium* and anti‐inflammatory *Bifidobacterium* spp. [161]. However, research on gut microbiota in ASD is challenging to evaluate due to nonhomogeneous results. Meta‐analyses show reduced Streptococcus and Bifidobacteria are associated with ASD diagnosis, while Actinobacteria phyla, Proteobacteria, Firmicutes, and Bacteroidetes are not [162]. It has been proposed that the interaction of gut bacteria and environmental variables influences the development of ASD (Figure 2). Glyphosate, an ineffective herbicide against *Clostridium botulinum* or *Clostridium perfringens* , can harm the human microbiome by killing sensitive Bifidobacterium and Lactobacillus and increasing Clostridium proliferation [163]. Environmental glyphosate increases *Clostridium* spp. in children with ASD and may be linked to sub‐acute *Clostridium tetani* infection [164]. The development of ASD is linked to the beta2‐toxin gene in the gut microbiome, produced by *Clostridium perfringens* [165]. Overuse of antibiotics can lead to the proliferation of Desulfovibrio bacteria, which are resistant to standard treatment, and the emission of LPS [166]. The development of behavior resembling ASD was attributed to prenatal exposure to LPS [167]. Animals with maternal immune activation exhibited microbial dysbiosis in the intestines, impaired GI barrier, elevated IL‐6 levels, and behaviors linked to ASD compared to the control group [168]. *Bacteroides fragilis* colonization reversed alterations, while *Lactobacillus reuteri* was able to alleviate intestinal inflammation caused by LPS [169].

These bacteria also stimulate the release of oxytocin, which positively influences anxiety and social behavior [168, 170]. The abnormal expression of the CLDN‐5 and CLDN‐12 genes, which encode TJ proteins, has been linked to GI problems in autistic children [171]. A study suggests that autistic symptoms in children with both ASD and GI problems may be linked to increased gut permeability levels [172]. Serotonin may be a crucial factor connecting gut microbiota with brain responses in ASD, as the most prevalent serotonin transporter‐based mutation is found in a mouse model [173]. In addition, patients with ASD have increased levels of gliadin and casein serum antibodies, which can cause autoimmune reactions [174], as well as increased phospho‐NF‐κB and inflammatory signaling [175]. Children with autism who experienced gastrointestinal issues exhibited higher numbers of CD8 and/or γδ T cells in the duodenum and colon [176, 177], and fewer peripheral T cells [178]. Monocytes, lymphocytes, eosinophils, and natural killer cells were found to have infiltrated the gut samples of autistic toddlers, indicating an inflammatory process [169]. In healthy individuals, the intestinal microbiota produces thiamine‐pyrophosphate, a cofactor for several enzymes, including transketolase, involved in the pentose phosphate oxidative pathway. Serum thiamine‐pyrophosphate concentrations are lower in autistic individuals [179], but neutrophils have higher amounts of ROS‐generating enzymes (NOX2/iNOS) [175]. Children with autism also revealed changed levels of metabolites in their urine. Antioxidant levels were decreased in urine samples obtained from those children [180]. Urine samples from children with autism showed greater levels of 3‐(3‐hydroxyphenyl)‐3‐hydroxypropionic acid than did samples from healthy controls [181]. Following oral therapy with vancomycin and Bifidobacterium supplements, elevated urine levels of HPHPA, 3‐hydroxyphenylacetic acid, and 3‐hydroxyhippuric acid observed in autistic children were considerably reduced. Following treatment, the patients showed less constipation and improved eye contact behavior [182]. Clostridiaceae metabolites like p‐cresol and p‐cresyl sulfate, along with elevated levels in urine and feces, are potential biomarkers for ASD. P‐cresol has an impact on noradrenaline production, dopamine metabolism, lipid peroxidation, and Na (+)‐K (+) ATPase activity in the brain. Increased gut permeability, gut infections, autistic behavior, and disease severity are all correlated with its concentrations [183].

A study [184] observed higher levels of indole and 3‐methylindole, [185] found higher concentrations of indolyl‐3‐acetic acid and indolyl‐lactate in the urine of autistic children. Well‐known indole producers include *Proteus vulgaris* , *Bacteroides* spp., *Clostridium* spp., *Escherichia coli* , *Paracolobactrum coliform*, and *Achromobacter liguefaciens* [184, 185]. Additionally, indole derivatives are metabolites of tryptophan, which is the precursor of serotonin, and in autistic patients, their physiological pathways are altered [185]. Isopropanol, a crucial metabolite linked to GI problems, is higher in the feces of autistic individuals [186]. Neurotransmitters can accumulate in the brain due to their metabolite, indoxylsulfate [185]. Children with ASD exhibit dysregulated metabolism of free amino acids, which are produced after proteins and peptides are hydrolyzed [180]. Autistic individuals have significantly higher fetal levels of free amino acids, which are consistent with the prevalence of proteolytic bacteria [187]. Maldigestion and malabsorption are problems for people with autism [188]. Autism‐related children may experience altered microbial burdens in their GI tracts due to impaired absorption of mono‐ and disaccharides, due to decreased expression of disaccharidases and hexose transporters. High concentrations of low‐molecular sugars in the large intestine stimulate the growth of fermenting bacteria, while hindering those that break down polysaccharides [189]. Vitamin B1 deficiency may be caused by reduced colonization of *Prevotella* spp., which break down plant polysaccharides and produce thiamine [160]. The presence of *Candida* spp. in the gut hinders the recolonization of commensal microbes [190].

Preclinical research indicates that rats given propionate intracerebroventricularly displayed behavioral and physiological signs of autistic ASD [191, 192]. Consuming items containing propionic acid or its precursors has been linked to more severe autistic behaviors in autistic individuals, in addition to GI symptoms. Gut bacteria, including *Clostridium* spp., *Bacteroides* spp., and *Desulfovibrio* spp., are strongly linked to ASD and produce propionic acid [193]. The condition of autistic individuals was improved by either consuming a diet free of propionic acid or using antibiotics to reduce the bacteria that produce it [194]. Additionally, 3‐nitropropionic acid, a mitochondrial neurotoxin, inhibits NADH synthesis in autistic individuals by forming when reactive nitrogen species interact with propionate [195].

A randomized clinical trial found that a mixture of bovine colostrum product and *Bifidobacterium infantis* significantly reduced the frequency of certain GI problems and atypical behavior in children with ASD [196]. A recent double‐blind, placebo‐controlled trial also showed that probiotics and prebiotics had positive benefits on autistic children [197]. 82.5% of 40 autistic children between the ages of 3 and 12 exhibited GI symptoms [198]. Additionally, ASD patients exhibit dysbiosis linked to the number of Verrucomicrobia, Fusobacteria, Bacteroidetes, and Firmicutes in their phylum, as well as the proportion of Firmicutes to Bacteroidetes [187]. Cryan and Dinan found that the modifications also influence the levels of volatile organic compounds (VOCs) and SCFAs in patients with ASD. Indole, a metabolite of tryptophan, is a precursor to melatonin and serotonin. The data should be interpreted cautiously due to the potential impact of antibiotic therapy or individualized nutrition on patients with ASD [199]. Studies on the gut‐brain axis reveal the ENS's crucial role in forming the gut‐brain axis, a vital communication pathway connecting the gut to the CNS. This axis influences emotions and behavior by promoting communication through immune responses, hormones, and neurotransmitters. Trillions of bacterial cells make up the human gut microbiome, which is vital to the gut‐brain axis's healthy operation. *Clostridium sporogenes* and *Bifidobacterium infantis* are beneficial bacteria that produce metabolites and neurotransmitters that regulate emotions and maintain neurological function. Pathogenic microbes like *Clostridium tetani* and *Clostridium bolteae* alter neurotransmitter function and behavior. They have been linked to GI problems and a higher severity and likelihood of ASD. Understanding the interactions between intestinal dysbiosis and NDs can provide crucial insights for diagnostic and treatment strategies. ASD is one of the many diseases linked to dysbiosis. This imbalance could significantly disrupt the host‐microbiota balance, potentially affecting the gut barrier and immune system. ASD patients may experience gut microbiota dysbiosis, which can affect their immune system, leading to changes in gut permeability and the release of proinflammatory substances. The “leaky gut” characteristic allows proinflammatory endotoxins like LPS to enter the bloodstream, altering CNS activity, influencing behavior, emotions, and neurodevelopment. Individuals with ASD exhibit alterations in certain microbial taxa, resulting in increased levels of beneficial bacteria like Proteobacteria, Lactobacillus, Bacteroides, and Clostridium. Gut microbial dysbiosis is linked to the pathophysiology of ASD, promoting gut barrier integrity, neurotransmitter synthesis, and immunological dysregulation [200].

### 4.4 Epilepsy

Over 65 million people worldwide suffer from epilepsy, a condition characterized by unprovoked, spontaneous seizures. Seizures are primarily caused by an imbalance between neuronal excitation and inhibition, regulated by glutamatergic and GABA‐ergic systems [201]. Epilepsy, or epileptogenesis, is a condition that develops after a causative event. Chronic epilepsy is a condition characterized by cellular and molecular changes that cause excitability in a normal neural network [202]. Epilepsy patients have two treatment options: nonpharmacologic interventions like surgery or ketogenic diet, and pharmacological treatments like antiseizure medications [201]. The ketogenic diet, which is low in carbs and high in fat, may be considered for those patients [203]. A patient with drug‐resistant epilepsy still experiences seizures after taking two carefully selected ASDs at the appropriate dosage, either alone or in combination with other ASDs [204]. A study investigating the gut microbiota composition of 42 resistant epileptic patients and 49 who responded to therapy revealed significant differences. Drug‐responsive patients' gut microbiota composition was comparable to that of healthy controls. Bifidobacteria and Lactobacillus were found to be more prevalent in patients with four or fewer annual seizures compared to those with more than four. Additionally, the drug‐resistant group had higher alpha‐diversity [205].

Lee et al. found that drug‐responsive patients and those who were drug‐resistant had different microbiota compositions but no variations in alpha or beta diversity [206]. Moreover, Olson et al. found that the anticonvulsant effect of a ketogenic diet was mediated by the microbiota in both the Kcna1−/− genetic model and the 6‐Hz induced seizure model. Antibiotic‐treated or GF‐treated mice exhibited no response to the seizure prevention effects of a ketogenic diet. Parabacteroides and *Akkermansia muciniphila* were more prevalent under the ketogenic diet. Furthermore, mice fed a control diet were protected against seizures by both treatment with Akkermansia and Parabacteroides and transplantation of ketogenic diet‐associated gut flora. Moreover, hippocampal GABA/glutamate ratios were associated with seizure prevention [207]. However, there is disagreement about whether the microbiota alterations brought on by a ketogenic diet are beneficial or harmful to people with epilepsy. Thirty healthy infants and fourteen drug‐resistant epileptic infants had different gut microbiome patterns. Most healthy newborns are Bacteroidetes, and a week of ketogenic diet improved 64% of children with drug‐resistant epilepsy, reducing seizures by 50%. The ketogenic diet led to an increase in Bacteroidetes and a decrease in Proteobacteria at the phylum level. Cronobacter declined while Prevotella, Bifidobacterium, and Bacteroides increased at the genus level [208].

Twenty kids with drug‐resistant epilepsy were included in a different trial. A 6‐month ketogenic diet resulted in varying levels of seizures in patients, with some experiencing a 90% or more decrease and others a 50%–89% reduction. A ketogenic diet led to increased Bacteroidetes in fecal microbial profiles, decreased Firmicute abundance, and decreased alpha diversity. Additionally, the nonresponsive group showed enrichment in Alistipes, Lachnospiraceae, Rikenellaceae, Clostridiales, and Ruminococcaceae [209]. Twelve children with epilepsy resistant to drugs who followed a ketogenic diet for 3 months showed no change in their alpha diversity compared to their nonketogenic counterparts. The intervention resulted in a decrease in the prevalence of Bifidobacteria, *Eubacterium rectale* , and Dialister. A ketogenic diet was found to increase the prevalence of *Escherichia coli* [210]. The ketogenic diet is utilized to treat GLUT1 deficiency syndrome, an early‐onset infantile epileptic encephalopathy caused by reduced glucose transport into the brain [211]. A pilot study found no significant differences between Firmicutes and Bacteroidetes, but an increase in *Desulfovibrio* spp., a group linked to inflammatory gut mucosa conditions caused by animal‐derived fat consumption [212]. FMT was successfully utilized to help a 17‐year‐old boy with Crohn's disease and epilepsy achieve remission of his neurological and intestinal symptoms. Despite discontinuing sodium valproate, the patient experienced no seizures during the 20‐month follow‐up period [212]. Six patients with drug‐resistant epilepsy experienced a brief cessation of seizures during antibiotic treatment. Antibiotics from several classes, including ciprofloxacin, clindamycin, azithromycin, clarithromycin, or piperacillin/tazobactam, amoxicillin, and amoxicillin/clavulanic acid, were used to treat the patients. The impact of macrolide antibiotics on ASDs was likely mediated by gut microbiota, despite potential increased serum concentrations enhancing their action [213]. However, Penicillin, an antibiotic, has been used in preclinical research to create a model of recurrent developing seizures [214].

In a pentylenetetrazole kindling animal model of epilepsy, a combination of probiotics [215], *Lactobacillus*, and *Bifidobacterium bifidum* had positive benefits [216]. Clinical studies were conducted on the variety and composition of microbiota in epilepsy. Epilepsy, a diverse group of diseases with varying etiologies, may have led to inconsistent outcomes due to methodological variations. Clinical studies involved individuals with drug‐resistant epilepsy, as participants were taking a variety of ASDs. The study found that not all drug‐resistant patients who began a ketogenic diet experienced improvement. Clinical investigations' control groups, whether from the same or separate households, may impact the microbiome profile and outcomes [205, 210].

### 4.5 Migraine

One of the most incapacitating diseases is migraine. Migraine is a significant global contributor to disability, particularly for individuals under 50 [217]. In the general population, 12% of people have migraines within a year. The prevalence of breast cancer in women is 18% per year and 33% per lifetime, while in men it is 6% per year and 13% per lifetime. Chronic ND is characterized by headache episodes and reversible neurological and systemic symptoms. Photophobia, phonophobia, cutaneous allodynia, and GI symptoms like nausea and vomiting are the most typical symptoms [218]. GI symptoms such as diarrhea or constipation can also occur. Migraines have been linked to GI conditions like inflammatory bowel disease [219]. Additionally, migraineurs had a considerably higher prevalence of *Helicobacter pylori* infection than controls (44.97% vs. 33.26%, respectively) [220]. Abdominal migraine, a pediatric functional abdominal pain disorder, is classified as a gut‐brain axis condition [221]. The “leaky gut” can lead to elevated levels of proinflammatory cytokines like TNF‐α, IL‐1β, and IL‐6, which can influence nociceptive responses and trigger migraine headaches [222]. Antibiotic treatment prolongs migraine‐like pain in wild‐type mice, but genetic deletion of TNF‐α or intraspinal injection of a TNF‐α receptor antagonist prevents pain prolongation. Antibiotic therapy prolonged Sp5C's TNF‐α upregulation, while probiotics reduced discomfort. GF mice had greater pain than WT mice, but gut colonization corrected pain enhancement. Gut microbiota dysbiosis increases TNF‐α levels in the trigeminal nociceptive system, leading to chronic migraine‐like pain [223].

A metagenome‐wide association analysis was conducted on 108 shotgun‐sequenced fecal samples from older migraine‐affected women and healthy controls. The migraine group displayed a significant reduction in alpha diversity while exhibiting a high enrichment of Firmicutes, particularly *Clostridium* spp. Healthy controls showed more advantageous bacteria such as *Methanobrevibacter smithii* , *Bifidobacterium adolescentis* , and *Faecalibacterium prausnitzii* [224]. Dysbiosis is linked to migraine duration and severity in young women without aura. Dysbiosis increases or decreases in microorganism diversity in stool samples [225].

### 4.6 Alzheimer's Disease

**FIGURE 3:**
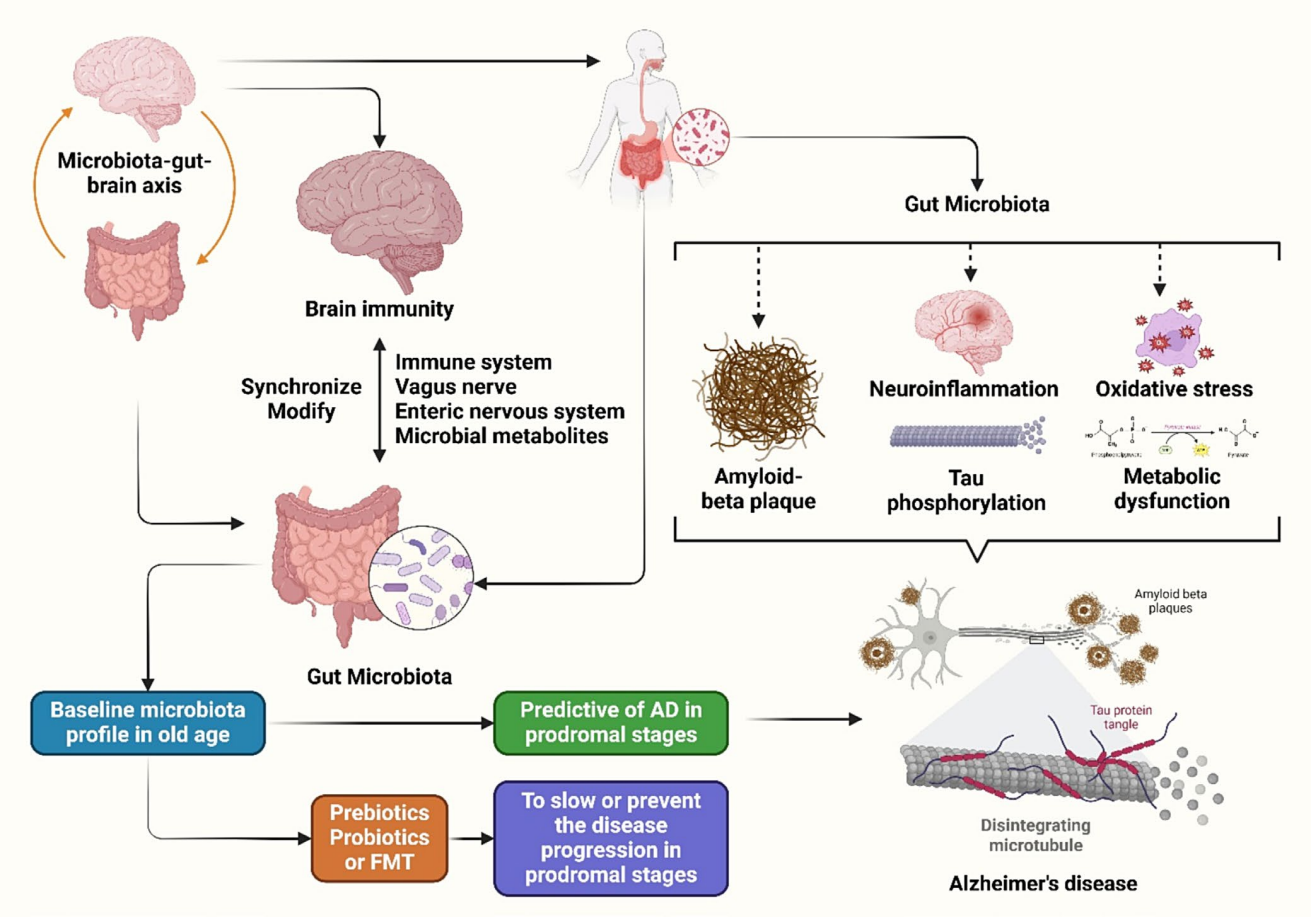
AD is influenced by hereditary and environmental factors, with the MGBA playing a major role.

The disease is characterized by a gradual decline in cholinergic functionality and the death of neurons in the cerebral cortex and hippocampal regions. The final condition is caused by monogenic gene mutations in presenilin (PRES 1 and PRES 2) and amyloid precursor protein (APP) [226]. Individuals carrying this mutation are likely to develop AD, characterized by intracellular neurofibrillary tangles and extracellular amyloid plaques [227]. AD is diagnosed based on specific features, primarily affecting significant neuron or synapse loss later in the disease [226, 228]. Pathogenic proteins activate glial cells in the brain, leading to inflammatory reactions that release nitric oxide, excitatory amino acids, inflammatory interleukins, and free radicals. These chemicals cause the death of neurons and their connections [229]. The pathogenesis of AD involves the excitatory neurotransmitter glutamate and the n‐methyl‐D‐aspartate (NMDA) receptor. NMDA receptors control memory and learning. NMDA receptor expression was found to be correlated with the gut microbiome. In a mouse model, the amount of NMDA receptors is dramatically decreased following the injection of antibiotics [230, 231]. Cyanobacteria can produce the neurotoxic β‐n‐methylamino‐L‐alanine and cause misfolding when improperly inserted into brain proteins. The risk of AD is increased by long‐term dietary exposure to the cyanobacterial toxin, which can cause neurofibrillary tangles and Aβ deposits in the brain [232, 233]. Neurotoxins like saxitoxin and anatoxin‐α, which are produced by other cyanobacteria, can accelerate the aging process [234]. Certain kinds of bacteria produce numerous neurotransmitters. γ‐aminobutyric acid (GABA) can be produced by *Bifidobacterium dentium* and *Lactobacillus brevis* . When these two phyla are reduced in the diet, the gut produces less GABA, which in turn causes the CNS to produce less GABA [235]. Gut microbes significantly influence the production of serotonin. Compared to a model animal with normal gut flora, the 5‐TH concentration was around 60% lower [236]. A brain‐derived neurotrophic factor (BDNF) molecule is produced in the brain and dispersed throughout the CNS. Numerous processes involve this protein. There is a decrease in both the brain and serum levels of BDNF. The gut microbiota, by controlling BDNF expression, may impact host cognition and potentially cause AD (Figure 3) [235].

Additionally, the gut bacteria are in charge of producing many vitamins, including vitamin B12. A higher incidence of AD and moderate cognitive impairment is linked to low serum levels of that vitamin [237]. There are additional potential pathophysiological pathways for AD. The first is because APP builds up in the stomach due to an increased Firmicutes/Bacteroides ratio. Aβ deposition increases in the early stages of sickness, starting in the stomach and confirmed in autopsies of AD patients [238]. Bacterial metabolites, including hydrogen, bile acids, trimethylamine n‐oxide (TMAO), and SCAFs, represent a second potential pathway. Host organisms use choline, betaine, and carnitine to produce TMAO. This metabolite uses host platelets to enter the brain, increasing β‐secretase activation and causing Aβ accumulation. Some bacteria form bile acids, which can enhance the permeability of the BBB. This allows cholesterol to build up in the brain and further increases the development of Aβ. Dysbiosis leads to AD pathogenesis by raising TMAO and amyloid levels while concurrently lowering specific advantageous components like hydrogen [237]. Neuroinflammation processes are caused by Gram‐negative bacteria that produce LPS. Amyloidosis and cognitive impairment are linked to the brain's pro‐inflammatory (*Schigella*/*Escherichia*) bacterial preponderance over anti‐inflammatory bacteria [238].

### 4.7 Parkinson's Disease

After AD, PD is the second most prevalent ND. The sixth decade of life is when this neurodegenerative movement condition first appears. Alfa‐synuclein (α‐syn) is the primary hallmark implicated in the disease [239]. Additionally, dopaminergic neurons in the substantia nigra pars compacta that project to the striatum are lost in PD [240]. PD symptoms include postural instability, muscle rigidity, resting tremor, and bradykinesia, while nonmotor symptoms include impairment of smell, autonomic dysfunction, mood or cognitive abnormalities, and sleep difficulties. These symptoms are attributed to progressive Lewy body development in brain regions [241]. α‐syn inclusions spread to other brain regions, eventually residing in the SN and cortex, causing damage through the olfactory tract or the dorsal motor nucleus of the vagus nerve (DMVN) [241, 242]. The direct insertion of α‐syn preformed fibril (PFF) into the muscular layers of the duodenum and pylorus initiates alfa‐syn aggregation and dopaminergic neuron degeneration [243]. Studies on vagotomy before and after injection suggest that the gut‐brain axis plays a role in the transmission of pathogenic alfa‐syn [244]. Furthermore, Braak et al. found that α‐syn disease originates in the ENS Meissner's plexus and progresses to the medulla oblongata via vagal preganglionic axons using retrograde axon transport [242]. Another study supported the theory by using various forms of alfa‐syn in rats and demonstrating that the vagal nerve transports them from the gut to the brain [245]. Microbiota changes were linked to higher fecal SCFA levels, specifically butyric acid, in both PD patients and healthy individuals. Bacteria producing less SCFA can lead to increased gut leakiness, colonic inflammation, and the potential for α‐syn depositions in the GIT [246, 247]. Numerous studies show microbial taxa altering even in the early stages of PD, based on fecal or mucosal specimens from PD patients and control samples. Notably, the most recent meta‐analysis of 15 case–control studies in PD populations from various geographic locations revealed some very intriguing results, including an increase in Christensenellaceae, Verrucomicrobiaceae, Ruminococcaceae, and Bifidobacteriaceae and a decrease in Lachnospiraceae, Prevotellaceae, and Faecalibacterium [248]. The symptoms are affected by these alterations in the gut flora. A decrease in ghrelin is linked to a low level of Prevotella and a higher level of Bifidobacterium and Lactobacillus. In the substantia nigra pars compacta, ghrelin has a role in controlling the activity of dopaminergic neurons. The acylated isoform of the drug shows neuroprotective properties on dopaminergic neurons in a mouse model of PD induced by MPTP. The PD population exhibits low levels of ghrelin content, regardless of the stage of the disease [249, 250, 251, 252]. Bacteroides is linked to the manufacture of riboflavin, while Prevotella is related to thiamine biosynthesis. In the early stages of PD, olfactory impairment is linked to low thiamine levels [253]. The quantity of this type of bacteria is considered a biomarker for disease. The production of SCFAs and anti‐inflammatory metabolites is a comparable function carried out by the Faecalibacterium family. The progression of PD shows a similar deterioration [254].

**FIGURE 4:**
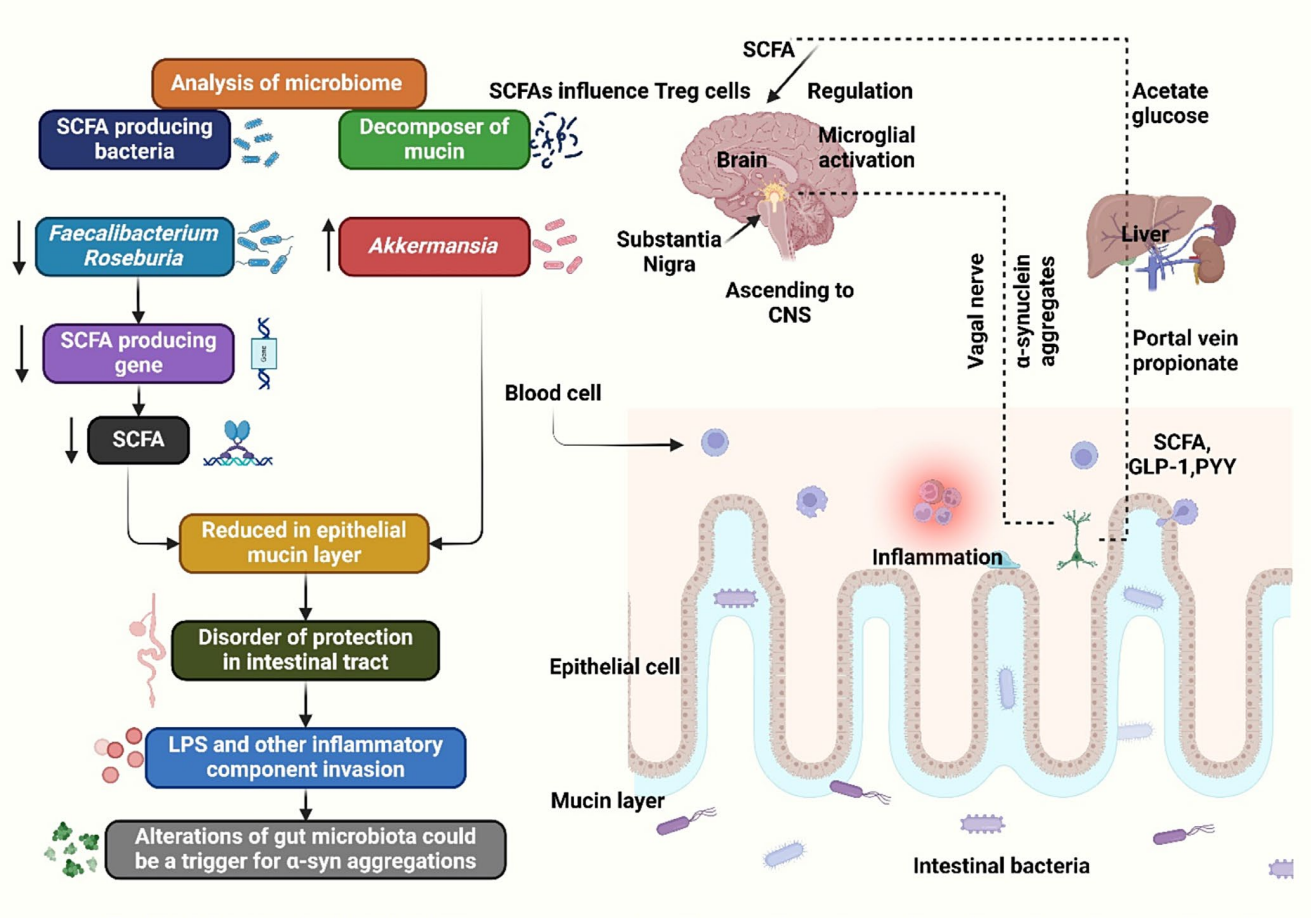
Gut dysbiosis contributes to PD by increasing intestinal permeability, reducing butyrate‐producing bacteria, and weakening the intestinal mucus layer. This leads to toxin exposure, aberrant α‐synuclein fibril aggregation, and neuroinflammation in the CNS.

However, a study found that the Bifidobacteriaceae family, which controls immunity and prevents the proliferation of harmful gut bacteria, is more prevalent in PD patients. Probiotics that break down carbohydrates and opportunistic infections are more prevalent than usual. PD reduces microorganisms that produce SCFAs (Figure 4) [255].

### 4.8 Multiple Sclerosis

MS, a chronic, inflammatory, demyelinating, and degenerative disease, impacts the CNS [256]. About 2.8 million people globally suffer from this autoimmune disease [257]. However, genetically predisposed individuals and those exposed to negative environmental conditions are susceptible to MS, but no significant differences in gut microbiota constitution were found between MS and control groups [258, 259]. In comparison to controls, only two investigations found that the MS population had higher levels of Metanobrevibacter and Akkermansia and lower levels of Prevotella [260], Bacteroides, and *Faecalibacterium prausnitzii* [258]. The MS model of EAE suggests that SCFAs may contribute to the onset and progression of the disease in mice. Moreover, transgenic mice from MS patients exhibited a higher frequency of brain autoimmunity posttransplantation compared to healthy individuals [261]. This disorder is more prevalent in young adults and women than men, potentially affecting people of any age. However, research indicates that autoimmune disease is the underlying pathophysiology of this condition, which may be influenced by hereditary and environmental factors [262].

Inflammatory plaques, which are concentrated regions of demyelination in the brain and spinal cord, are the main pathogenic features of MS. Neuronal loss results from this inflammation's destruction of oligodendrocytes and myelin [263]. MS is a disease mediated by CD4 T cells, with the major histocompatibility complex class II locus being the most significant genetic risk factor [264]. Antigen‐presenting cells, including dendritic, macrophage, and B cells, express MHC Class II molecules, activating CD4+ T cells and initiating the adaptive immune response [265]. The human leukocyte antigen, a major MHC, is a crucial site linked to the risk of MS. The MHC class II region significantly influences the immunological response. Interactions with CD4+ (helper) T cells are one of its functions [266]. Furthermore, IFN‐γ coordinates several defense mechanisms that strengthen immune responses. These include enhancing antigen presentation, promoting the activation of macrophages, facilitating antiviral and antibacterial immunity, and regulating cellular proliferation and apoptosis [267]. Nonetheless, IFN‐γ's effect on MS has been contradictory, with some research linking it to a pathogenic and pro‐inflammatory role. Interferon‐gamma, a cytokine that activates macrophages and enhances their enzyme secretion, is produced by CD4+ T helper 1 (Th1) cells. Additionally, IFN‐γ causes the generation of reactive oxygen and nitrogen intermediates, which exacerbate nearby tissue damage. Th17 cells generate cytokines such as IL‐17, IL‐21, and IL‐22, which are linked to the development of the disease and the inflammatory response [268]. Teriflunomide and fingolimod are oral medications used to treat relapse types of MS, primarily affecting CD4+ T cells, reducing the lymphocyte count in peripheral blood [269]. This procedure is crucial for maintaining healthy neurotransmission and protecting the brain from excitotoxicity and glutamate‐induced harm. Studies in EAE show that loss of end‐feet around small blood vessels is linked to impaired BBB function, high permeability, and CNS inflammation in MS models [270]. The manufacture of multiple inflammatory cytokines and chemokines by inflammatory T cells activates local glial cells, which leads to demyelination, axonal loss, and BBB disruption [271]. Inflammation is stimulated, and the release of pro‐inflammatory cytokines, including IL‐1β, IL‐6, IL‐12, IL‐17, IL‐23, and TNF‐α, aids MS progression [272]. By influencing tight junctions, endothelial cells, and microglial phagocytosis, astrocytes' TGF‐β, IL‐10, and IL‐27 secretion can regulate immune cells' ability to cross the BBB [273].

Astrocytes release chemicals that hinder remyelination, preventing oligodendrocyte precursor cells from maturing into myelinating oligodendrocytes [274]. The variety and composition of the microbiota can significantly impact the progression and development of MS pathogenesis and other immunological disorders. Poor microbial populations of Haemophilus, Sutterella, Adlercreutzia, Coprobacillus, Lactobacillus, Clostridium, Anaerostipes, Faecalibacterium, Prevotella, Bacteroides, and Parabacteroides are found in the microbiota of MS patients [256]. MS affects various microorganisms, causing specific changes and their effects on the illness. Commensal microbes can stimulate inflammatory (Th1 or Th17) and regulatory (Th2) responses. Microbes play a crucial role in controlling immunological responses, affecting the pathophysiology of immune‐mediated diseases like MS, highlighting their complex function [275]. The normal gut microbiota usually regulates *Clostridium difficile* 's growth, preventing disease development. *Clostridium difficile* causes severe cases of enterocolitis, causing symptoms like diarrhea, abdominal discomfort, and colon inflammation in individuals without underlying disease. Dysbiosis in the gut microbiota in MS patients may lead to an excess of *Clostridium difficile* , causing increased inflammation and worsening MS symptoms. The Clostridium class includes specific bacterial groups called Clostridium XIVa and IV clusters. These bacteria support gut health and immunological function and are a normal component of the gut microbiota in healthy people. MS patients show a decrease in Clostridium XIVa and IV species, suggesting a possible dysbiosis that could impact the condition's pathophysiology. The two main phyla of bacteria in the gut microbiota are Firmicutes and Bacteroidetes. Gut health and overall well‐being are linked to a balanced ratio of Bacteroidetes and Firmicutes in healthy individuals. These changes may impact synaptic function, neuroplasticity, and neuroinflammation in MS. A pilot study was conducted to evaluate the impact of vitamin D and glatiramer acetate therapy on the gut flora of MS patients and healthy controls [276].

Vitamin D is a crucial immunomodulatory substance that plays a vital role in various immunological functions in both innate and adaptive immune systems. The substance directly impacts T cells, affecting their growth and operation, and indirectly alters the activity of other immune cells that interact with T cells. Vitamin D's diverse functions underscore its importance in maintaining immunological homeostasis and its potential link to immune‐mediated diseases. The study involved healthy white women with vitamin D deficiency, regardless of their relapsing–remitting MS. Patients were either treated with Glatiramer acetate, an immunomodulator used to treat and control MS, or left untreated. The abundance of operational taxonomic units was measured through 16S rRNA hybridization to a DNA microarray. Despite significant gut bacterial ecosystem overlap, MS patients show less prevalence of operational taxonomic units like Faecalibacterium compared to healthy controls. Patients with MS who received glatiramer acetate showed a distinct gut microbiota composition compared to those who did not receive treatment. The Lactobacillaceae, Ruminococcus, Bacteroidaceae, Faecalibacterium, Clostridium, and other Clostridiales exhibited this. Additionally, after taking vitamin D supplements, untreated MS patients had higher levels of Akkermansia, Faecalibacterium, and Coprococcus than other groups. The pilot study suggests that therapeutic interventions like glatiramer acetate and vitamin D supplements may affect the gut microbiota composition, potentially affecting the pathophysiology and management of MS. *Akkermansia municipalis* and other bacterial and archaeal taxa have increased in the gut microbiota of MS patients [261, 277]. Another trial included 68 controls and 64 MS patients not receiving treatment. It was discovered that MS patients had higher levels of the bacteria *Acinetobacter calcoaceticus* and *Akkermansia muciniphila* , whereas controls had higher levels of *Parabacteroides diastonis*. Clinical in vitro research has shown that *Acinetobacter calcoaceticus* promotes the differentiation of T helper cells (Th1 and Th2) and inhibits the differentiation of regulatory Treg. *Akkermansia muciniphila* promotes Th1 cell growth. Tr1, which secretes IL‐10, was generated by *Parabacteroides diastonis*. MS‐associated microbiota alterations affect T lymphocyte differentiation, requiring future research on microbes' role in adaptive autoimmune responses. Enterobacteriaceae populations are higher in MS patients compared to healthy controls [7].

A 13‐year‐old research study involving 18 relapsing–remitting MS cases and 17 controls was conducted. MS patients often experience short‐term disease, and half of them do not receive immunomodulatory medications to treat their condition. Bacteria from the Desulfovibrionaceae family (including Bilophila and Desulfovibrio) and Christensenellaceae were more abundant in MS cases than in controls, but Lachnospiraceae and Ruminococcaceae were less abundant. Furthermore, MS patients exhibit a higher prevalence of microbial genes related to glutathione metabolism, suggesting that environmental factors may influence gut microbiota colonization in these patients [278].

### 4.9 Obstructive Sleep Apnea

Obstructive sleep apnea (OSA) is a condition where the upper airway collapses during sleep, reducing or stopping ventilation. The person experiencing breathing restoration experiences hypoxia, hypercapnia due to airway obstruction, and increased sleep awakenings, often linked to specific events [279]. Type 2 diabetes, arterial hypertension, stroke, cardiac arrhythmias, coronary diseases, and cognitive changes are all associated with obstructive sleep apnea [280, 281]. An increase in anaerobic microbes in the gut may result from the OSA‐related intermittent hypoxia. Dysbiosis in individuals with OSA is linked to cardiometabolic, neurobehavioral, and gastrointestinal abnormalities, as well as increased tissue permeability, inflammation, and OS. People who snore showed a drop in Actinobacteria/Proteobacteria and a rise in Firmicutes/Bacteroidetes, suggesting that snoring may impact the gut microbiota. Intermittent hypoxia in children with OSA causes an increase in intestinal barrier inflammation and a decrease in the diversity of the intestinal flora [282]. Gut dysbiosis was noted in patients with OSA in studies that assessed their gut flora [283]. Experimental research shows that probiotics and prebiotics may help with this dysbiosis [279, 281]. When used with weight loss, prebiotics and probiotics improve quality of life and reduce cortisol, interleukin 6 (IL‐6), visceral fat, liver fat, and abdominal circumference [281].

### 4.10 Sleep Deprivation

Sleep deprivation occurs when an individual receives less sleep than the necessary amount for relaxation and good health. Cardiovascular diseases, atherosclerosis, coronary diseases, autonomic nervous system changes, immunological changes, metabolic disorders, mood, cognitive, attention, memory changes, and stroke are some of the pathologies it can cause or contribute to. Additionally, it is linked to increased adiposity, insulin resistance, and appetite [284]. Stressors like sleep deprivation or fragmentation can change the gut microbiota, causing dysbiosis and increasing intestinal permeability, which can increase inflammation [285]. Gut microbiome diversity was reduced following acute sleep deprivation [281, 286]. Lack of sleep has been shown to induce intestinal cell damage and OS in animal models [287]. Microbiome diversity was linked to both wakefulness following sleep start and sleep efficiency. Research shows a positive correlation between IL‐6, a cytokine linked to sleep issues, and overall microbiome diversity, with sleep‐deprived individuals having fewer Bacteroidetes and higher Firmicutes concentrations. This alteration is linked to obesity and may contribute to weight gain in individuals who lack sufficient sleep [288]. Insomnia is another sleep disorder linked to shorter sleep duration. Both acute and chronic sleeplessness may be linked to a decrease in bacterial diversity. Anaerobic bacteria were reduced in patients with the disease. Chronic insomnia patients have a unique microbiome structure, potentially leading to a decrease in butyrate‐producing microbes and an increase in inflammatory markers. The species Blautia increased, potentially linked to cognitive diseases like diabetes and GI disorders, and a decrease in Faecalibacterium [289].

### 4.11 Bipolar Disorder

Bipolar disorder (BD), a chronic and recurrent illness with specific symptoms similar to SCZ, is another severe mental disease [290, 291]. Over 1% of the global population suffers from BD, a severe and recurrent neuropsychiatric condition that ranks 17th globally after anxiety and depressive disorders [292]. Factors affecting brain health include basic brain changes, pathophysiological causes, oxidative and nitrosative stress, calcium and neurotrophin signaling pathways, and cellular bioenergetic changes [293]. BD patients may suffer from depression and manic symptoms or hypermanic symptoms. Bipolar disorder is characterized by extreme low mood, hopelessness, discontent, and lack of interest in life, or high mood, happy thoughts, and minimal sleep needs. Symptoms of these conditions lead to a reduced quality of life, mental or functional impairments, and a high risk of suicide [294]. Numerous studies show a direct correlation between disposition, cognitive disturbance, and the constantly changing gut microbiota [295]. Patients with BD exhibit higher inflammation, possibly indicating disorders, due to increased bacterial translocation markers from the intestinal lumen compared to healthy individuals [290, 296]. Obesity and metabolic disorders are frequently linked with BD, enhancing the prognosis and complicating the treatment of the condition [297, 298]. Currently, mood stabilizers like lithium, valproate, carbamazepine, lamotrigine, olanzapine, and fluoxetine are utilized to treat individuals with BD pharmacologically. These medications alter the gut microbial ecosystem [290, 294]. A study on the stool microbiome revealed significant differences between individuals with BD and healthy individuals [299]. The discovery of the autochthonous gut bacterium Faecalibacterium, which has been linked to diseases and depressive states, was significant. The exact amount of Faecalibacterium was discovered [293].

A study [300] found that BD individuals had four times less Clostridiaceae than healthy controls. Clostridiaceae and Collinsella are responsible for the fermentation of carbohydrates and the production of SCFA [301]. Clostridiaceae are crucial for gut barrier integrity, while an increased number of Lactobacilli is believed to contribute to obesity in BD [293, 302]. Low Bifidobacterium counts in patients with BD and MDD negatively impact stress response, while higher Lactobacillus levels may be beneficial for sleep disturbances [303, 304]. BD patients exhibited lower TRYCAT levels in their serum/plasma or cerebrospinal fluid (CSF) compared to healthy controls [305]. The altered gut microbiota composition in BD patients suggests a correlation between GM dysbiosis and the progression of the disease [306]. Studies investigating the diversity of the intestinal microbiome reveal that higher levels of Coriobacteriaceae are linked to elevated cholesterol levels [307]. Higher levels of Lactobacilli have been linked to the development of obesity and BD [302]. Diseases can also be linked to low levels of the autochthonous gut bacterium Faecalibacterium [293]. In BD patients, four times more Clostridiaceae were involved in the fermentation of carbohydrates, producing SCFAs, than in the control group [300, 301].

### 4.12 Dementia

Dementia is a psychological condition characterized by memory issues, personality changes, a decline in thinking and social skills, and difficulty reasoning. These symptoms affect patients, families, and society physically, psychologically, socially, and economically. Dementia currently affects 50 million older people worldwide, and by 2050, that number is projected to rise to almost 130 million people [308]. Dementia, a condition characterized by agitation, abnormal motor behavior, fear, joy, sadness, indifference, a lack of inhibition, delusions, hallucinations, and changes in eating or sleep, is a psychological and behavioral manifestation [309]. Randomized clinical trials aim to reduce risk factors like unhealthy eating habits, excessive drinking, smoking, diabetes, depression, genetics, family aggregation, high blood pressure, obesity, and others in older adults [308]. Changes in the Bacteroides population directly affect the decrease or stimulation of risk factors for cognitive losses like dementia [310, 311]. Lactobacillus, Bifidobacterium, and Streptococcus are probiotic strains that enhance human health by boosting the immune system, acting as a barrier against pathogens, and producing antimicrobial metabolites [311]. Lactobacillus and Bifidobacterium are believed to protect against dementia by enhancing neurotransmitter activity, particularly acetylcholine synthesis, which is linked to cognitive acquisition and retention mechanisms [311, 312, 313]. A study has shown a connection between dementia and chemicals linked to the gut microbiota. The study on fecal samples from 82 dementia‐free and 25 dementia patients found increased concentrations of metabolites, including ammonia, indole, p‐cresol, and phenol, which are significant risk factors for AD [313].

### 4.13 Attention‐Deficit Hyperactivity Disorder

ADHD is a condition characterized by impulsivity, hyperactivity, and attention issues, affecting various behaviors and cognitive repertoires, and causing delayed maturation in children [314, 315, 316]. The shrinkage of different areas of ADHD brains is often linked to a decrease in synaptic density, rather than a loss of neurons [317]. ADHD children show delayed cortical maturation, particularly in the lateral PFC, which regulates working memory, reward evaluation, attention direction, and inappropriate responses [315, 318]. Children with ADHD have a motor cortex that reaches peak development 4 months earlier than control children, potentially leading to impulsivity [315]. The primary etiological elements of this disease are thought to be the genes for dopamine and serotonin transmitters and the dopamine receptors DRD4 and DRD5 [319]. A microbiome study found Actinobacteria, including Bifidobacterium, have a higher prevalence in ADHD cases, while Firmicutes have a lower abundance [320]. Another study found that individuals with ADHD had higher levels of Blautia as compared to healthy controls, indicating an association between intestinal microbiota and ADHD [321]. The research on the relationship between ADHD and GBMA should consider a broader range of demographics [322]. Probiotic supplementation in early childhood may reduce neuropsychiatric diseases, while early injection of *Lactobacillus rhamnosus* GG may decrease ADHD risk [323]. It was demonstrated that *L. rhamnosus* controlled central GABA receptor expression and emotional behavior in a mouse via the vagus nerve [324]. Dietary habits affecting ADHD individuals should be considered, with artificial color additives being a significant factor in reducing hyperactivity [325]. The intake of omega‐3 PUFAs, including docosahexaenoic acid and eicosapentaenoic acid, is crucial for proper neurotransmission, receptor activation, and membrane fluidity [326].

A study on animal male ADHD models revealed that individuals with a diet high in omega‐3 PUFAs showed improved concentration and reduced impulsivity [327]. Through the MGBA, the gut microbiota has been linked to the pathophysiological processes of ADHD. Changes in the MGBA cause OS and neuroinflammation, which in turn cause the basic symptoms of ADHD and related comorbidities such as sleep disorders. Research indicates that acetaminophen, a common analgesic and antipyretic, and maternal stress may increase the risk of ADHD in children during pregnancy [328]. Research indicates that probiotics may have a therapeutic benefit for kids with ADHD. Probiotic supplements have demonstrated increasing outcomes in terms of lowering symptoms of ADHD and enhancing cognitive performance [323]. The gut microbiota may play a role in the pathophysiology of ADHD, potentially influencing dopaminergic metabolic pathways in individuals with the condition [326]. The omega‐3 to omega‐6 polyunsaturated fatty acid ratio can lead to neuroinflammation and dopaminergic dysfunction, exacerbating ADHD symptoms. Glutamic acid decarboxylase requires pyridoxal phosphate as a cofactor, the active form of vitamin B6. Glutamic acid decarboxylase breaks down tryptophan, a precursor to serotonin, xanthurenic acid, and kynurenic acid, transforming glutamate into the inhibitory neurotransmitter GABA. Research on tryptophan metabolism suggests that the biochemical abnormalities in ADHD are primarily caused by B6 deficits [329]. SCFA production is a well‐known activity of bacterial species like Bacteroides and Clostridium. SCFAs may have an impact on ADHD symptoms by influencing neurogenesis, OS, and neuroinflammation [330]. The Bacteroidaceae family was shown to be more prevalent in teenagers with ADHD [331]. Additionally, Wang et al. found an augmentation of specific Bacteroides species in the ADHD cohort. It found no significant differences in beta diversity between groups, but large variability in beta diversity and decreased alpha diversity within the ADHD group [332].

## 5 Conclusion and Future Perspectives

MGBA, a crucial regulator of neurological and neuropsychiatric diseases, links gut microbial composition to brain health. Research links gut dysbiosis to neurotransmitter imbalances, OS, and neuroinflammation, leading to conditions like ASD, depression, AD, and PD. Gut microbiota's importance in mental health is confirmed. Furthermore, microbiome‐targeted therapeutics, including probiotics, prebiotics, and dietary changes, offer promising prospects for disease prevention and therapy. Nevertheless, despite the growing amount of data, clinical translation remains limited, necessitating further research into customized microbiome‐based treatments. Future studies should focus on developing microbiome sequencing tools to identify patient‐specific microbial fingerprints for targeted therapies. Clinical trials are conducted to assess the effectiveness and safety of microbiome‐based treatments for neurological and neuropsychiatric diseases, ensuring thorough monitoring and therapeutic validation. Multi‐omics integration uses proteomics, metabolomics, and genomics to understand the intricate relationships within MGBA and their impact on brain function. The review explores the effects of environmental factors, physical activity, and diet on the gut microbiome, aiming to improve brain health. Next‐generation microbiome therapeutics explores advanced treatments like postbiotics, probiotics, and medications targeting the MGBA for neuroprotection.

## Author Contributions

Md. Faysal: conceptualization; data curation; formal analysis; investigation; methodology; resources; software; supervision; visualization; roles/writing – original draft; writing – review and editing. Mehrukh Zehravi: data curation; investigation; methodology; resources; software; roles/writing – original draft; writing – review and editing; supervision. Baishakhi Sutradhar: data curation; investigation; methodology; resources; software; roles/writing – original draft; writing – review and editing. Md Al Amin: data curation; investigation; methodology; resources; software; roles/writing – original draft; writing – review and editing. Thukani Sathanantham Shanmugarajan: formal analysis; investigation; validation; visualization; roles/writing – review and editing. Uppuluri Varuna Naga Venkata Arjun: formal analysis; investigation; validation; visualization; roles/writing – review and editing. Susithra Ethiraj: formal analysis; investigation; validation; visualization; roles/writing – review and editing. Akiladevi Durairaj: formal analysis; investigation; validation; visualization; roles/writing – review and editing. Girija Dayalan: formal analysis; investigation; validation; visualization; roles/writing – review and editing. Shaik Khadeer Ahamad: formal analysis; investigation; validation; visualization; roles/writing – review and editing. Safia Obaidur Rab: formal analysis; investigation; validation; visualization; roles/writing – review and editing. Kannan Raman: formal analysis; investigation; validation; visualization; roles/writing – review and editing. Talha Bin Emran: conceptualization; formal analysis; funding acquisition; investigation; methodology; project administration; supervision; validation; visualization; writing – review and editing.

## Ethics Statement

The authors have nothing to report.

## Consent

The authors have nothing to report.

## Conflicts of Interest

The authors declare no conflicts of interest.

## Data Availability

The data that support the findings of this study are available from the corresponding author upon reasonable request.
